# Invasive Physiological Assessment of Lower Limb Peripheral Arterial Disease: A Narrative Review

**DOI:** 10.3390/jcdd12020077

**Published:** 2025-02-18

**Authors:** Sarosh Khan, Samer Fawaz, Uzma Sajjad, Christopher Cook, Grigoris V. Karamasis, John Davies, Ali Kordzadeh, Elafra Nour, Alun H. Davies, Ankur Thapar, Thomas Keeble

**Affiliations:** 1 Essex Cardiothoracic Centre, Basildon SS16 5NL, Essex, UK; sarosh.khan2@nhs.net (S.K.); uzma.sajjad1@nhs.net (U.S.);; 2 Anglia Ruskin School of Medicine & MTRC, Anglia Ruskin University, Chelmsford CM1 1SQ, Essex, UKa.thapar@nhs.net (A.T.); 3 Attikon University Hospital, School of Medicine, National and Kapodistrian University of Athens, 10679 Athens, Greece; 4 Mid and South Essex Vascular Unit, Basildon SS16 5NL, Essex, UK; 5 Section of Vascular Surgery, Department of Surgery and Cancer, Imperial College London, London SW7 2AZ, UK

**Keywords:** peripheral arterial disease, endovascular therapy, physiology

## Abstract

Peripheral arterial disease (PAD) affects over 236 million people globally, with endovascular treatment as the predominant mode of revascularization. While pre-procedural lesion assessment typically relies on non-invasive Doppler measurement, invasive physiological assessment offers a promising approach to guide lesion selection and provide real-time evaluation of angioplasty success. This review explores the current methods, challenges, and future directions of invasive physiological assessment in PAD. Sensor-tipped wires, particularly pressure sensor-tipped wires (pressure-wires), enable precise evaluation of stenoses through indices such as peripheral fractional flow reserve (pFFR) measured during hyperaemia. pFFR can identify significant flow-limiting lesions, assess angioplasty efficacy, and predict tissue healing. Additional indices, including Doppler-wire derived flow reserves and resistance measurements, further enhance the understanding of lesion physiology. Early data support the utility of these techniques for guiding treatment decisions, although the variability in methodologies highlights the need for standardization and outcome-driven cut-off values. This review uniquely consolidates evidence on invasive physiological assessment in PAD, addressing critical gaps and providing a framework for future research. By advancing lesion-specific evaluation and procedural optimization, this work underscores the transformative potential of these techniques in improving patient outcomes and redefining PAD management.

## 1. Background

Peripheral arterial disease (PAD) affects 236 million adults world-wide [1]. In the UK, most vascular treatments are for chronic limb-threatening ischemia (CLTI) and involve endovascular therapy (EVT) [2]. Evidently, endovascular therapy (EVT) is the most common mode of treatment for CLTI in the UK.

Surprisingly, the intra-procedural haemodynamic assessment of peripheral vascular lesions has seen limited clinical utilisation. Assessment of the haemodynamic effect of angiographic borderline stenoses (typically one in a series of lesions) is currently performed pre-procedurally using Doppler velocity measurement and signal waveform. This has practical limitations, such as the inability to view through calcium (tibials) or bowel gas (iliacs), difficulty assessing serial stenoses close together and no consideration of the microvascular resistance in the foot, which will determine wound healing. Doppler measurement is also cumbersome to perform during angioplasty and therefore pragmatically not often used to guide the operators. Finally, when considering vessels that spontaneously markedly change in size (e.g., in the venous system), a physiological assessment may be more appropriate than an anatomical assessment alone.

The use of invasive physiology has changed clinical practice over the last decade in interventional cardiology. Coronary physiological assessment allows for the identification of appropriate lesions for angioplasty [3], thereby also providing safe deferral of borderline stenoses that appear visually significant at first in two-dimensional angiography [4]. Considering PAD, physiological assessment may guide the treatment of a proximal lesion alone, i.e., the treatment of the superficial femoral artery, rather than combined with tibial angioplasty, thereby exposing the patient and team to less risk, radiation and contrast. Furthermore, invasive physiology not only considers the impact of stenoses in the macrovasculature (named arteries) but also the microvasculature (muscle and skin capillary bed) post-revascularisation. This is important because without an effective microvasculature, any impact on wound healing in CLTI will be poor. In the coronary field, intra-procedural physiology has also been used to predict the efficacy of angioplasty, thus leading to physiologically guided treatment [5]. A physiology-driven approach to PAD may help tailor treatment, for example, if the final prognosis may be considered to be so poor due to high microvascular resistance that no large vessel revascularisation should be attempted in the first place. This may identify patients who would benefit from early amputation and would support decision making by providing objective haemodynamic data to discuss with patients. Currently, intra-procedural assessment of stenoses by catheter-derived or pressure-wire-derived gradients are not included in guidelines for PAD [1]. The differences between coronary and peripheral blood flow, particularly the former occurring predominantly during diastole and the latter during systole, as well as the frequent occurrence of serial stenoses within the peripheral circulation and the impact of multiple branching and collateral circulations on haemodynamic and tissue perfusion, cannot be overlooked. Furthermore, the distinct anatomical and pathological features of peripheral artery disease (PAD), including differences in plaque composition and distribution across the aorta, large iliac, femoral, and below-the-knee arteries, underscore the need for tailored approaches [6]. These features, which contrast with coronary artery territories, reinforce the importance of adapting technologies to the unique challenges posed by the peripheral circulation.

This narrative review on the invasive physiological assessment of lower limb PAD aims to focus on the different methods undertaken to physiologically assess the macrovasculature and also briefly discuss the more nascent field of microvasculature physiological assessment. In doing so, this review aims to address significant gaps in the use of invasive physiological assessments for PAD. By synthesizing current evidence, it highlights the potential to refine lesion selection, improve procedural outcomes, and establish a foundation for future research in this emerging field.

### Literature Search Methodology

The literature search was conducted using PubMed, Medline, Scopus and Embase databases from inception to December 2024. Keywords included “peripheral arterial disease”, “physiological assessment”, “fractional flow reserve”, “stenosis assessment” and “hyperaemia”. Inclusion criteria focused on studies investigating invasive physiological techniques in normal and diseased human lower limbs. Studies were excluded if they lacked quantitative physiological data, utilised non-invasive physiological studies, focused on the assessment of stenosis with imaging or measured physiology in limbs other than lower limbs. Only articles in English were included.

## 2. Methods of Invasive Physiological Assessments in Lower Limbs

The initial invasive assessment of pressure gradients was conducted utilising fluid-filled 4 or 5Fr catheters that were passed distal to the lesion and compared the distal pressure to the proximal unobstructed aortic pressure. A mean arterial pressure (MAP) gradient of 10 mmHg or greater was considered a haemodynamically significant stenosis [7]. The main drawback of this method was that the catheter would occlude the stenosis itself, therefore providing a falsely higher gradient. Conversely, specially designed wires with a pressure sensor installed within them (pressure wires) were accurately able to measure gradients and predict stenosis diameter as they did not have such a haemodynamic influence [8]. Therefore, the pressure wire, with its considerably smaller profile, is considered a better tool for assessing lesions invasively and has largely replaced catheter-based pressure measurements.

Other wire-based assessments can be performed with alternative sensors that have been manufactured to allow for measurements intravascularly. Key wire technologies and their associated measured indices that have been demonstrated within the literature are summarised below and in Table 1.

### 2.1. Pressure Sensor-Tipped Wires

The placement of pressure sensor-tipped wires across gradients allows for measurements of pressures across stenoses during the cardiac cycle. The peak (systolic) pressure gradient or mean pressure gradient can be measured [7,9]. The challenge with measuring absolute gradients is the influence of systemic blood pressure changes that occur during anaesthesia or due to the use of drugs such as heparin which induce vasodilation. The beat-to-beat changes in peak systolic pressure can be significant and variable, and therefore, measuring a mean gradient provides a more reproducible and consistent measurement that still is dependent on systolic pressure.

**Figure 1:**
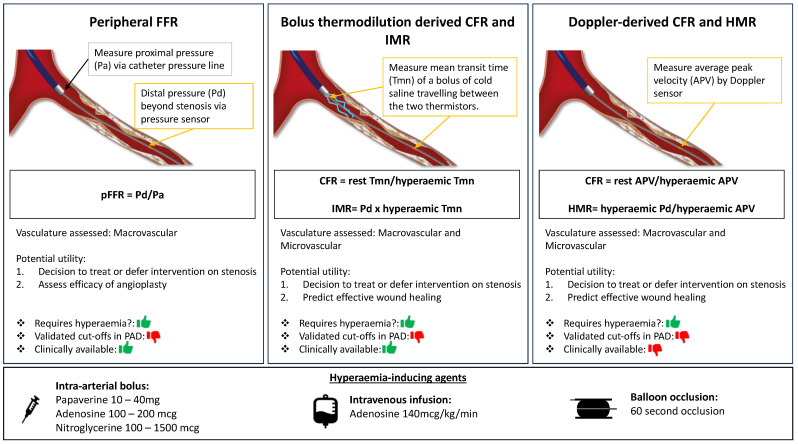
**Central Illustration**. Wire-based invasive physiology indexes in peripheral arterial disease. Illustration of wire-based invasive physiology indexes in peripheral arterial disease assessment. Left panel: peripheral fractional flow reserve (pFFR) using pressure sensor wire. Middle panel: bolus thermodilution derived coronary/peripheral flow reserve (CFR) and index of microcirculatory resistance (IMR) measured using a thermistor and pressure sensor tipped wires. Right panel: doppler wire derived CFR and hyperaemic microvascular resistance (HMR). pFFR evaluates the macrovasculature only whilst bolus and doppler derived indexes assess both macrovasculature and microvasculature. All three indexes rely on induction of hyperaemia and different methods of achieving hyperaemia are shown.

The assessment of an arterial stenosis as a ratio of mean distal (outflow) arterial pressure (Pd) to proximal (inflow) vessel pressure (Pa) overcomes some of the problems of changes in inflow systolic pressure, such as during anaesthesia. A resting assessment is called Pd/Pa, while during hyperaemia, it is termed peripheral fractional flow reserve (pFFR). Hyperaemia is achieved by using an agent to induce maximal vasodilatation and thereby minimising microvascular resistance. Many different agents have been used (see Table 2 and the Figure 1).

In order to measure such a ratio, a dedicated 0.014″ pressure sensor-tipped wire is first passed into the proximal vessel where it is equalised to Pa. The Pa can be transduced via the sheath side port or via the catheter. The wire is then passed beyond the stenosis. Once a stable trace is achieved, the Pd is obtained. The resting ratio of Pd/Pa can be calculated. To obtain the pFFR, a hyperaemic agent is given either through the sheath as an intra-arterial bolus or intravenously as an infusion. Once a steady state of hyperaemia is achieved, the pFFR value is obtained. There are commercially available pressure wires that are licensed for use within peripheral arteries.

### 2.2. Temperature Sensor-Tipped Wires

An alternative method of measuring a surrogate of blood flow is from thermodilution. Within the coronary arteries, this can be assessed with bolus thermodilution by measuring transit times of boluses of normal saline. Comparing flow at baseline to hyperaemia can allow for the calculation of coronary flow reserve (CFR). A pressure and temperature sensor located at the distal tip of the pressure wire is equalised at the ostium of the sheath and then subsequently passed beyond the lesion. Subsequently, thermodilution assessment is undertaken by bolus injections of 3 mL of saline at room temperature from the sheath in the same manner described for assessing coronary flow reserve [23]. The temperature sensor detects the change in mixed-blood temperature, and the transit time is acquired from injection to detection. The mean transit time (Tmn) is inversely proportional to arterial blood flow [23].

### 2.3. Doppler Sensor-Tipped Wired

In addition to a pressure sensor, a Doppler sensor-tipped wire can be used to assess mean pressure and peak velocity distal to the lesion. An average of three consecutive heart beats provides the average peak velocity (APV). The APV is acquired at rest and during hyperaemia and therefore can also provide a CFR value using bolus thermodilution, like with temperature sensor-tipped wires. Doppler wires are predominantly used within research settings and not clinically available.

## 3. Methods of Inducing Hyperaemia

Indices requiring measurements during hyperaemia require agents to reliably induce this phenomenon. Hyperaemia reduces microvascular resistance, allowing for the assessment of stenosis severity under conditions of maximal flow. This provides a more accurate evaluation of functional ischemia compared to resting (non-hyperaemic) pressure measurements [24]. There are a variety of agents that can be used to induce hyperaemia within the published literature; however, for the purpose of this review, we have identified the most frequently used agents within the literature. Intra-arterial papaverine at a dose of 30 mg has been shown to induce hyperaemia reliably when assessing SFA lesions [15]. However, there was no difference in pFFR with papaverine doses of 10 mg, 20 mg and 30 mg in a mix of iliac and SFA vessels [18]; similarly, Hioki et al. [12] found papaverine 20 mg sufficient compared to 25 mg and 30 mg in only iliac arteries. This suggests that different vessels may require different doses to generate sufficient hyperaemia and provide reliable pFFR values, which is similar to differing intra-coronary doses when measuring fractional flow (FFR) measurements in the left or right coronary artery [25].

Intra-arterial adenosine has been effectively used to generate pFFR gradients; however, the doses used have been variable [10,11,22]. These can range from one hundred micrograms to a weight-adjusted one hundred forty micrograms per kilogram. Similarly, intravenous adenosine infusion of 140 mcg/kg/min has also been utilised. This variability highlights how a standardized protocol for inducing hyperaemia is necessary, including any adjustments for vessel assessment. Although the effects on peripheral vessels have not been quantified, extrapolating from coronary artery assessment, papaverine may be the preferred choice as a bolus hyperaemic agent over adenosine given its longer duration of stable hyperaemia (44 vs. 12 s) [25,26]. Furthermore, the perceived risk from papaverine-induced QT prolongation and arrhythmia would be theoretically lower due to peripheral injection and the side effects are less frequent than the side effects associated with IV adenosine [26]. The utilisation of other medications like nicorandil shows some promise compared to papaverine; however, there is a need for significantly larger sample sizes to demonstrate their safety [27].

An alternative to chemically induced hyperaemia is reactive hyperaemia which can be generated by balloon occlusion of the artery during angioplasty. Within the coronary arteries, balloon occlusion was used to induce hyperaemia prior to the adoption of chemical agents [28,29]. Recent research has identified that sixty seconds of balloon occlusion of a coronary artery can produce hyperaemia that is equivalent to intra-coronary papaverine or intravenous adenosine [30]. Accordingly, the role of balloon occlusion needs to be further explored within the peripheral vessels to identify a standardized method of inducing hyperaemia; however, this must be mitigated by the risks associated with damage to normal vasculature and may be more useful after an angioplasty procedure for assessing its efficacy.

Although papaverine appears to be safe and can reliably produce pFFR values, the data are from limited sized studies and thus need further reproducibility in a wide variety of vessels to be proposed as the agent of choice. Comparative studies with alternative agents should also be pursued at the same time. The absence of an established hyperaemic agent hinders the future adoption and utilisation of physiological indices dependent on these agents.

## 4. Clinical Studies Evaluating Macro and Microvascular Physiological Techniques in Lower Limbs

### Peripheral Fractional Flow Reserve (pFFR)

The pFFR or the ratio of proximal to distal pressure through a stenosis is the most reported method of assessing peripheral lesions invasively. Albayati et al. assessed 41 patients with iliac and superficial femoral stenoses utilising te pFFR with intra-arterial boluses of adenosine. They demonstrated that the diameter of the stenosis according to CT corresponded with a worsening pFFR and that the pFFR also correlated strongly with calf oxygenation measured by CMR. Finally, a greater increase in the FFR signalled resolution of symptoms and signs but an FFR post-PTA > 0.74 predicted successful revascularisation. A similar study evaluated 48 SFA stenoses using bolus papaverine for hyperaemia. They identified that at doses of 20, 30 and 40 mg, papaverine led to a pFFR of 0.87 ± 0.10, 0.84 ± 0.10 and 0.84 ± 0.10, respectively, with a baseline non-hyperaemic pFFR of 0.97 ± 0.04. Although the pFFR with 20 mg and 30 mg was not significantly different (*p* = 0.602), the values of pFFR with 30 and 40 mg were the same (*p* = 1.00). Therefore, both adenosine and papaverine can be used to create hyperaemia and generate pFFR values effectively. Banerjee et al. [10] were the first to report an association between TLG and pre/post exercise ABPI and walking distance in 19 patients with superficial femoral artery (SFA) disease. They used both intra-arterial adenosine and nitro-glycerine to induce hyperaemia and showed strong correlation between pre-operative and intra-operative haemodynamic assessment. They also compared the pFFR to the TLG (with TLG being the difference between proximal and distal pressure, and the pFFR being expressed as the ratio of distal to proximal pressure during hyperaemia) and found a strong correlation.

Hioki et al. also demonstrated similar findings between post-exercise ABPI and pFFR in 16 patients with isolated iliac artery lesions and papaverine-induced hyperaemia [12]. A significant and linear correlation between post-exercise ABI and pFFR during hyperaemia was identified (r = 0.857, *p* < 0.001). Furthermore, they identified that this correlation was stronger than that with peak-to-peak pressure gradient during hyperaemia, thus demonstrating that the pFFR, which is a mean pressure gradient measurement, is a more accurate method for physiological assessment.

However, Kobayashi et al. performed the first validation of papaverine for pFFR in humans utilising incremental doses [15]. They measured the pFFR in 48 (44 successful) SFA lesions. A contralateral approach for measuring pFFR was undertaken with equalisation of pressure in the infrarenal aorta. Bolus papaverine doses of 20, 30 and 40 mg were injected into the ostium of the common iliac artery. They identified that at doses of 20, 30 and 40 mg, papaverine led to a pFFR of 0.87 ± 0.10, 0.84 ± 0.10 and 0.84 ± 0.10, respectively, with a baseline non-hyperaemic pFFR of 0.97 ± 0.04. Although the pFFR with 20 mg and 30 mg was not significantly different (*p* = 0.602), the values of pFFR with 30 and 40 mg were the same (*p* = 1.00). Furthermore, the results remained the same when analysed by the number of run-off vessels.

Lotfi et al. were the first to compare intra-procedural pFFR and peak systolic velocity measured pre-operatively with DUS in 20 patients with SFA lesions and adenosine-induced hyperaemia [11]. A post-intervention pFFR < 0.95 was significantly associated with increasing peak systolic velocity and suggested residual stenosis. Similarly, Murata et al. demonstrated that isosorbide dinitrate-induced hyperaemia for pFFR calculation correlated well with pre-procedural peak systolic velocity in 22 patients with iliofemoral stenosis [13]. In addition, the authors suggested that the optimal cut-off value for pFFR as an indicator of hemodynamic significance was 0.85.

## 5. Doppler-Derived APV

Ikeoka et al. [19] demonstrated an increment in baseline and hyperaemic APV from before EVT compared with after EVT in SFAs. From baseline and hyperaemic APV values, the velocity flow reserve (hyperaemic APV/baseline APV) can be derived. In this same study, velocity flow reserve was not correlated with the percentage area of stenosis prior to EVT; however, the pFFR measured with the pressure wire component of the same wire demonstrated a better correlation (r = 0.140 vs. −0.716).

The utilisation and clinical uptake of Doppler wires in the coronary space has been limited due to expense, the availability of these wires and also significant noise on the Doppler signal, relegating utility mainly as a research tool [31].

An alternative method of assessing stenosis severity utilising a Doppler wire is to derive hyperaemic stenosis resistance (hSR). This is performed using a Doppler wire and is calculated by the mean pressure difference divided by the APV during hyperaemia. Ikeoka et al. [19] demonstrated that hSR was highly correlated with >75% area stenosis of SFA lesions. Furthermore, they identified an hSR of >0.36 to be an optimum cut-off value to predict this degree of stenosis.

## 6. Peripheral Flow Reserves

The ability of the subtended territory of an interrogated vessel to augment flow from rest to hyperaemia is termed the flow reserve or vasodilatory capacity. As described above, in coronary physiological assessment, this can be termed CFR. Miki et al. [14] investigated the change in mean transit time in normal SFAs after variable doses of papaverine (10, 20, 30 and 40 mg) were given intra-arterially with a 3 min wash-out period in between. They calculated the percent increase in blood flow (%IBF) as the average resting Tmn divided by the hyperaemic Tmn for each dose. They identified that the mean %IBF for 10, 20, 30 and 40 mg was 240%, 250%, 495% and 434%, respectively. Therefore, 30 mg papaverine was considered sufficient to cause maximal hyperaemia. Furthermore, there appeared to be good reproducibility of the measurements at this dosage.

Although this was the first demonstration of what is the peripheral equivalent of coronary flow reserve (CFR), the assessed values had a significant range between patients in this small sample size of patients. However, this study was conducted in normal arteries; therefore, large variations in flow reserve could be expected given the large flow within peripheral arteries during systole.

## 7. Peripheral Arterial Microvascular Assessment

All of the above indices and measurements are measurement surrogates of flow via changes in pressure, velocity or transit times of fluid boluses. Blood flow within a vessel is also regulated by the resistance in the tissue bed mediated via the microcirculation. This is termed microvascular resistance. Although the flow reserves measured by bolus thermodilution or Doppler wires make a combined assessment of both the large interrogated artery and the microvasculature, a more dedicated understanding of microvascular resistance may be helpful, especially in regard to wound healing.

There are different methods used to assess coronary microvascular resistance that use temperature or Doppler wires, as described above. The index of microcirculatory resistance (IMR) is derived during assessment by bolus thermodilution flow reserve (peripheral CFR equivalent), as described by Miki et al. The IMR calculation takes the distal pressure in the vessel beyond the stenosis (Pd) multiplied by the mean transit time (Tmn) during hyperaemia.

Miki et al. [14] described this method of utilising bolus thermodilution to derive an equivalent to coronary flow reserve but in SFA lesions. However, during this assessment, the IMR is automatically calculated, which the authors presented in their figures but did not include in their publication. Given that the IMR is calculated based on transit time, the increasing doses of papaverine led to lower transit times and, as a result, lower IMR’s in their validation of papaverine-induced hyperaemia.

Microvascular assessment remains a field that is still incompletely understood and is an evolving frontier. The current methods of assessing microvascular function are derived from those used to assess the microvasculature and suffer from the same weaknesses.

## 8. Utility in Procedural Efficacy

The utility of invasive physiological assessments to guide decision making within the peripheral intervention sphere has mainly been explored using pressure-wire-based techniques.

Murata et al. proposed a pFFR of 0.85 to be haemodynamically significant when compared to the peak systolic velocity ratio measured by DUS [13]. Ikeoka et al. [16] demonstrated that a pFFR < 0.88 (and hSR < 0.36) could predict 75% area stenosis measured by intravascular ultrasound (IVUS). However, the former evaluated iliofemoral vessels and the latter the SFA. Furthermore, the respective hyperaemic agents were intra-arterial nitrate and papaverine, which may contribute to unequal levels of hyperaemia. There is likely a threshold, which may be vessel-specific, that can be applied to the assessment of moderate-severity stenoses to help decide on the need to revascularize akin to an FFR < 0.80 utilised within coronary vessels [3].

After revascularisation, post-intervention physiology may provide insights into the effects of recoil, residual stenosis, dissection flaps and negative prognostic outcomes. A mean pressure gradient <10 mmHg after angioplasty has been quoted as a good haemodynamic marker suggesting no further requirement for stenting. However, without a hyperaemic agent to minimise peripheral resistance, this is not as reliable compared to pFFR.

In a prospective non-randomised study [15], 48 patients with 51 SFA lesions treated with EVT had pFFR measurements performed after the procedure. At 12 months, the patients with residual/restenosis had a significantly lower pFFR post-EVT, and a cut-off of ≤0.92 provided optimal predictive power. Using this value, vessels with a pFFR > 0.92 compared to ≤0.92 had residual/restenosis rates of 4.5% and 35.7%, respectively. In another observational study [11], patients with a pFFR < 0.95 post-EVT of SFA stenosis had a significantly more rapid rise in peak systolic velocity measured by duplex, which would be associated with restenosis.

## 9. Utility in Patient Outcomes

Peripheral angioplasty is offered for intermittent claudication or for limb salvage, and therefore, any procedural physiological assessment should be safe, be suitable for day-case use and provide a positive clinical impact. Currently, there are limited data beyond case series. In one series, four patients had infra-popliteal artery EVT with the pFFR measured before and after treatment. Two patients with a post-EVT pFFR ≥ 0.90 had wounds that healed swiftly and two patients with a post-EVT pFFR < 0.90 had no wound healing [21]. Similarly, Nakamura et al. [20] describe a case of common femoral artery stenosis in which peak systolic velocity and post-exercise ABPIs were not diagnostic of PAD but pFFR improved from 0.86 to 0.99 post-EVT. The patients’ ABPI continued to improve at 1- and 3-month follow-ups without recurrence of symptoms.

The Figure 1 shows a summary of the main wire-based techniques used for the assessment of peripheral lower extremity arterial disease and their potential benefit in procedural and patient outcomes.

Clearly, further evaluation of the impact of intravascular physiological assessment of stenoses in peripheral vessels on patient outcomes is required.

## 10. Conclusions

This review uniquely consolidates fragmented evidence in an emerging field, providing a roadmap for standardizing and validating invasive physiological assessments in PAD. These techniques hold significant potential for optimizing lesion selection, enhancing procedural outcomes and improving patient care. The integration of different physiological techniques offers promising avenues for accurately assessing both macrovascular and microvascular aspects of peripheral arterial blood flow and resistance.

However, significant limitations, including variability in methodologies, small sample sizes and a lack of validation, hinder widespread clinical adoption. Future research should focus on standardizing techniques, validating thresholds for clinical decision making and integrating complementary invasive modalities like IVUS. A physiology-driven approach has the potential to enhance lesion selection, improve procedural outcomes and provide objective data to guide clinical decisions whilst operating. Such advancements could ultimately lead to better prognostic outcomes for patients with PAD.

## 11. Future Directions

Future research must prioritize large-scale trials to validate these techniques, establish standardized protocols and evaluate their clinical efficacy. The development of operator-independent and vessel-specific assessment methods, such as those emerging from coronary physiology, holds promise in addressing many of the current challenges [32]. For example, the currently enrolling IMPACT-PAD study [ClinicalTrials.gov:NCT05035771] aims to evaluate the feasibility of a novel approach using continuous thermodilution to measure absolute blood flow and resistance within PAD in humans. Furthermore, the identification of factors such as tandem or serial lesions and their impact on different physiological techniques will be important to ensure appropriate lesion-specific selection of physiological tools.

Addressing these procedural challenges through rigorous reproducibility and efficacy studies could pave the way for intra-procedural physiological assessment, coupled with IVUS and non-invasive perfusion metrics (e.g., transcutaneous oxygen tension), to become a robust and holistic cornerstone of effective PAD assessment and management.

## Figures and Tables

**Table 1:** Indexes associated with invasive wire-based assessment of physiology.

Index	Equipment	Measures	Potential Utility	Hyperaemia Required?	Notes on Limitations
Trans-stenotic pressure ratio (Pd/Pa)	Pressure- wire	Ratio of distal pressure beyond stenosis (Pd) compared to central pressure (Pa) at rest (i.e., no hyperaemia) during whole cardiac cycle.	Stenosis severity	No	Impaired discriminatory ability to identify significant versus non-significant lesions due to variability during systole and diastole. Variable microvascular resistance in absence of hyperaemic agent.
Peripheral fractional flow reserve (pFFR)	Pressure- wire	Ratio of distal pressure beyond stenosis compared to central (aortic or main vessel) pressure during maximal hyperaemia during whole cardiac cycle.	Stenosis severity Efficacy of angioplasty	Yes	Unclear impact of serial lesions on measurements.
Average peak velocity (APV)	Doppler wire	Doppler peak flow velocity average over >3 heartbeats (APV) at rest.	Stenosis severity	Yes	Impaired discriminatory ability to identify significant stenosis due to influence of systemic pressures and variable microvascular resistance. Doppler wire signal can be impaired due to noise. Doppler wire limited to research use.
Coronary flow reserve (CFR)	Thermistor-pressure tipped wire	Mean transit time of 3 ml cold saline boluses between two thermistors on wire. Ratio of measurement at rest to hyperaemic measurement to calculate PFR.	Large vessel and microvascular assessment	Yes	Operator-dependent and variability with injection of cold boluses. Unclear volume required for peripheral bolus to ensure reproducible signal to calculate transit time.
	Doppler wire	Doppler peak flow velocity average over >3 heartbeats (APV) at rest and during hyperaemia.			Doppler wire signal can be impaired due to noise. Doppler wire limited to research use.
Hyperaemic stenosis resistance (hSR)	Doppler wire	Ratio of pressure gradient (hyperaemic proximal pressure—hyperaemic proximal pressure) to hyperaemic APV.	Stenosis severity	Yes	Limitations of Doppler wire as above.
Index of microcirculatory resistance (IMR)	Thermistor- pressure tipped wire	Measured at same time as bolus CFR. Calculated by multiplying hyperaemic Tmn by distal pressure (Pd).	Microvascular assessment	Yes	Influenced by stenosis in large vessel and otherwise requires correction.
Hyperaemic microvascular resistance (HMR)	Doppler wire	Measured at same time as Doppler CFR. Calculated by dividing hyperaemic mean distal pressure by hyperaemic APV.	Microvascular assessment	Yes	Limitations of Doppler wire as above.

Indexes are established from coronary research; therefore, limitations are derived from coronary experience, or the theoretical basis of the techniques as applied within coronary circulation.

**Table 2:** Published articles on use of invasive physiology to assess culprit vessels.

Author	N	Syndrome	Vessel	Index Assessed	Equipment/Procedure	Hyperaemic Agent	Findings
**Garcia et al. [8]** **(2007)**	16	PVD	Iliac, subclavian, renal artery and 1 aorta	CPG (catheter-pressure gradient) PWG (pressure-wire gradient)	Catheter-based 4 or 5 Fr and 1 Fr larger sheath 0.014-inch pressure wire	IA Nitro-glycerine 200 mcg	Only included if baseline gradient was <10 mmHg then went on to have hyperaemia. MAP gradient with CPG always higher than PWG. CPG of 10 corresponded to diameter stenosis of 50%, but PWG of 68%.
**Banerjee et al. [10]** **(2011)**	19	IC	Superficial femoral artery	TLG (trans-lesional gradient) “pFFR”	0.014-inch pressure wire	IA Adenosine 100 and 200 mcg IA ISDN 100 and 200 mcg Calf cuff inflation	All TLG measurements correlated well with exercise ABI (Spearman’s rho >0.850) including FFR. TLG using 100 mcg Adenosine correlated best with ABI ≤0.70 with greatest AUC; a TLG cut-off of 11 mmgHg with a sensitivity of 71.43% and a specificity of 100%.
**Lotfi et al. [11]** **(2012)**	20	IC	Superficial femoral artery	pFFR vs. DUS PSV pre and post	0.014-inch pressure wire Equalisation in proximal SFA vessel	IA Adenosine 1 mg/kg	Resting pFFR (Pd/Pa) and pFFR were significantly different. Baseline PSV and pFFR correlated well (−0.77, *p* < 0.001). pFFR < 0.95 post-EVT of SFA stenosis had a significantly more rapid rise in PSV by DUS.
**Hioki et al. [12]** **(2014)**	16	IC	Iliac artery	pFFR	0.014-inch pressure wire Equalisation in proximal iliac vessel	IA Papaverine 20 mg, 25 mg and 30 mg.	Established papaverine 20 mg after using 20, 25 and 30 mg validation. No significant difference in pFFR at higher doses. Prospective study of pFFR with rest and post-exercise ABIs, with the severity of lesions confirmed by Doppler US. pFFR lower in positive exercise ABI group than in negative group. Mean MAP showed no difference in positive vs. negative exercise ABI groups.
**Murata et al. [13]** **(2015)**	23	IC & CLTI	Iliofemoral	Mean pressure gradient (MPG = mean aortic pressure—mean distal pressure) and mean pressure ratio (MPR = pFFR = mean distal pressure/mean aortic pressure)	0.014-inch pressure wire	IA ISDN 250 mcg	Hyperaemic MPR (pFFR) and hyperaemic MPG were both correlated with PSVR on DUS. Optimal cut-off value for pFFR as an indicator of haemodynamic significance (PSVR > 2.5) was 0.85.
**Miki et al. [14]** **(2015)**	12	Normal vessels	Superficial femoral artery	Bolus thermodilution derived mean transit time (Tmn) and percentage increase in blood flow (%IBF): Tmn rest/Tmn hyperaemia	0.014-inch pressure wire and equalisation 3 cm distal from ostium of SFA	IA Papaverine 20 mg, 30 mg and 40 mg intra-arterial	Papaverine 30 mg was sufficient to induce maximal hyperaemia. Reproducible measurements.
**Kobayashi et al. [15]** **(2016)**	48	IC & CLTI	Superficial femoral artery	pFFR after EVT	0.014-inch pressure wire Equalisation at descending aorta above iliac bifurcation Contralateral femoral crossover approach	IA Papaverine 20 mg, 30 mg and 40 mg intra-arterial	Successful pFFR in 44/48 patients. Papaverine 30 mg was sufficient to induce hyperaemia.
**Kobayashi et al. [15]** **(2016)**	48	IC & CLTI	Superficial femoral artery	Mean pFFR and systolic pFFR pre and post EVT	As above	IA Papaverine 30 mg	pFFR can be used to predict restenosis. Post-stenting pFFR was significantly lower in the restenosis group. Best mean pFFR cut-off post-stenting to predict restenosis was 0.92 (sensitivity 0.64, specificity 0.91).
**Ikeoka et al. [16]** **(2018)**	32	IC	Superficial femoral artery	FFR, VFR (H-MR (hyperaemic mean distal pressure/hyperaemic APV)	0.014-inch pressure/Doppler Equalised in CFA Contralateral approach	IA Papaverine 20 mg	FFR and VFR improved significantly but h-MR did not after EVT. A high h-MR prior to EVT may predict improvement in h-MR reduction after EVT.
**Ruzsa et al. [17]** **(2018)**	39	CLTI	CLTI; Below the knee arteries; Popliteal, anterior tibial, peroneal, tibiofibular and posterior tibial	Pd/Pa, pFFR pre and post EVT	0.014-inch pressure wire	IA Papaverine 40 mg	Before and after EVT comparison. Resting Pd/Pa improved and pFFR improved. Pd/Pa and pFFR both weakly correlated (r −0.31) with % area stenosis. Pd/Pa and FFR did not show correlation with DUS measurements. At 1 year, MAE’s and MACCE in 7 (17.9%) and 9 (23.1%) of patients.
**Maeda et al. [18]** **(2018)**	26	IC & CLTI	14 iliac arteries; 12 superficial femoral arteries	pFFR	0.014-inch pressure wire Ipsilateral or contralateral approach	IA Adenosine 100 mcg bolus And either (1) 200 mcg Adenosine (2) 10 mg, 20 and 30 mg Papaverine (3) 1.5 mg ISDN	Identified no difference in pFFR with papaverine 10, 20 or 30 mg. Therefore, considered 10 mg Papaverine sufficient to induce maximal hyperaemia. IA 100 mcg adenosine as effective as 200 mcg Adenosine, 10 mg Papaverine or 1.5 mg ISDN in inducing hyperaemia to generate a hyperaemic Pd/Pa i.e., pFFR.
**Ikeoka et al. [19]** **(2019)**	24	IC	Superficial femoral artery	pFFR, VFR, H-SR (hyperaemic stenosis resistance) pre and post EVT	0.014-inch pressure/Doppler wire Equalised in CFA Contralateral approach	IA Papaverine 20 mg	pFFR and h-SR and VFR improved significantly from pre- to post-EVT. Lesion severity, measured by IVUS, was strongly associated with h-SR and FFR, but not VFR, before EVT. h-SR > 0.36 and pFFR < 0.88 were cut-off values to predict over 75% area stenosis.
**Nakamura et al. [20]** **(2022)**	1	IC	Common femoral artery	pFFR pre and post EVT	OmniWire, Philips, 0.014-inch RCFA puncture for left CFA catheterisation. Equalisation: left external iliac artery	40 mg Papaverine and 1 mcg Alprostadil intra-arterial	PSV and exercise–stress ABI were not diagnostic of PAD in patient with intermittent claudication. USS demonstrated significant lesion and PSV 230 cm/s. Patient had pFFR that was 0.86 and had EVT. Post-EVT, pFFR improved to 0.99. PSV remained similar at 227 cm/s. ABI continued to improve at 1-month and 3-month follow-up without recurrence of symptoms.
**Ahmed et al. [21]** **(2022)**	4	CLTI	Infra-popliteal arteries	pFFR	0.014-inch pressure wire Contralateral approach; pressure wire delivered distal with microcatheter that remains in-situ; equalisation of pressure SFA or popliteal artery.	IV Adenosine 140 μg/kg/min infusion for 3 min	Resting “FFR” (i.e., Pd/Pa) and FFR improved in all 4 cases of CLTI from pre-procedure to post-procedure. The two patients with post-EVT FFR ≥ 0.90 had angiographically equivocal results yet the swiftest wound healing. Patients who had post-PTA FFR ≤ 90 had no wound healing.
**Albayati et al. [22]** **(2024)**	41	IC & CLTI	Femoral and iliac (also normal iliac)	pFFR vs. DUS (PSV), CT diameter stenosis	0.014-inch pressure and Doppler wire; contralateral approach for femoral; for iliac, contralateral for aortic pressure & ipsilateral femoral for distal pressure.	IA Adenosine 100 mcg/kg bolus distal to lesion via microcatheter subsequently withdrawn	Baseline pFFR values decreased with incremental percentage diameter stenosis. A lower pFFR was associated with critical limb threatening ischaemia. Larger increase in pFFR after successful PTA vs. unsuccessful. A post-PTA pFFR >0.74 predicted successful revascularisation.

ABI: ankle brachial index; CFA: common femoral artery; CLTI: chronic limb-threatening ischaemia; DUS: duplex ultrasound; EVT: endovascular therapy; h-SR: hyperaemic stenosis resistance; SFA: superficial femoral artery; IC: intermittent claudication; IVUS: intravascular ultrasound; Pa: proximal/aortic pressure; Pd: distal pressure; pFFR: peripheral fractional flow reserve (each manuscript may have used different nomenclature for same metric); PSV: peak systolic velocity; PVD: peripheral vascular disease; VFR: vascular flow reserve.

## Glossary

%IBF	percentage increase in blood flow
EVT	endovascular therapy
PAD	peripheral arterial disease
Pa	proximal vessel (inflow) arterial pressure
Pd	distal vessel (outflow) arterial pressure
pFFR	peripheral fractional flow reserve (ratio of Pd/Pa during hyperaemia)
SFA	superficial femoral artery
TLG	translesion pressure gradient (Pd-Pa before hyperaemia)
